# Activation of P2X7 and P2Y11 purinergic receptors inhibits migration and normalizes tumor-derived endothelial cells *via* cAMP signaling

**DOI:** 10.1038/srep32602

**Published:** 2016-09-02

**Authors:** D. Avanzato, T. Genova, A. Fiorio Pla, M. Bernardini, S. Bianco, B. Bussolati, D. Mancardi, E. Giraudo, F. Maione, P. Cassoni, I. Castellano, L. Munaron

**Affiliations:** 1 Department of Life Sciences & Systems Biology, University of Torino, Torino, Italy; 2 Nanostructured Interfaces and Surfaces Centre of Excellence (NIS), University of Torino, Torino, Italy; 3 Dept. of Molecular Biotechnology and Health Sciences, University of Torino, Torino, Italy; 4 Department of Clinical and Biological Sciences, University of Torino, Torino, Italy; 5 Candiolo Cancer Research Center, Torino, Italy; 6 Department of Medical Sciences, Torino, Italy

## Abstract

Purinergic signaling is involved in inflammation and cancer. Extracellular ATP accumulates in tumor interstitium, reaching hundreds micromolar concentrations, but its functional role on tumor vasculature and endothelium is unknown. Here we show that high ATP doses (>20 μM) strongly inhibit migration of endothelial cells from human breast carcinoma (BTEC), but not of normal human microvascular EC. Lower doses (1–10 mm result ineffective. The anti-migratory activity is associated with cytoskeleton remodeling and is significantly prevented by hypoxia. Pharmacological and molecular evidences suggest a major role for P2X7R and P2Y11R in ATP-mediated inhibition of TEC migration: selective activation of these purinergic receptors by BzATP mimics the anti-migratory effect of ATP, which is in turn impaired by their pharmacological or molecular silencing. Downstream pathway includes calcium-dependent Adenilyl Cyclase 10 (AC10) recruitment, cAMP release and EPAC-1 activation. Notably, high ATP enhances TEC-mediated attraction of human pericytes, leading to a decrease of endothelial permeability, a hallmark of vessel normalization. Finally, we provide the first evidence of *in vivo* P2X7R expression in blood vessels of murine and human breast carcinoma. In conclusion, we have identified a purinergic pathway selectively acting as an antiangiogenic and normalizing signal for human tumor-derived vascular endothelium.

Extracellular adenosine 5′-trisphosphate (ATP) is released in the vascular lumen in response to sympathetic nerves stimulation or to changes in blood flow and control several vascular functions^1^,^2^,^3^,^4^,^5^,^6^. Moreover, it plays pivotal roles in more trophic and long-term processes of vascular remodeling such as atherosclerosis, restenosis and angiogenesis, mediated by endothelial cell (EC) migration, proliferation, differentiation and death. The biological actions and total amount of ATP in the extracellular medium depend on the balance between release by different cell types and breakdown into ADP and Adenosine by ectonucleotidases, as well as on the pattern of purinergic receptors expressed by target cells^1^,^2^,^3^,^7^. Purinergic receptors are usually classified into P1R (binding to Adenosine) and P2R types (recognizing ATP, ADP and UTP). P2R family includes P2X ligand-gated ion channels and G proteins-coupled P2Y receptors^8^. The seven known P2XR subtypes (P2 X 1–7) are hetero- or homotrimeric calcium-permeable cation-conducting channels which are expressed in both arteries and veins^8^,^9^,^10^. On the other hand, P2YR can be divided on the basis of their endogenous agonists into adenine nucleotide-preferring (P2Y1, P2Y11, P2Y12 and P2Y13) and uracil nucleotide-preferring (P2Y2, P2Y4, P2Y6 and P2Y14) subtypes. In normal ECs, P2Y1, P2Y2 and P2Y6 are predominant and mediate endothelium-dependent vasodilation *via* the synthesis and release of prostacyclins and nitric oxide (NO)^3^,^11^,^12^,^13^. Moreover, P2YR recruitment results in PLCβ activation and InsP3 production, followed by the release of Ca^2+^ from the ER^14^,^15^. In addition to Ca^2+^ signaling, some P2YRs (mainly P2Y11) and P2XRs promote cAMP increase that regulates endothelial barrier stabilization^16^,^17^.

During inflammatory processes and cancer, ATP and other nucleotides accumulate in tissue microenvironment to reach concentrations much higher than those measured in healthy tissues^8^,^18^,^19^,^20^. In tumors, extracellular ATP origins from necrotic and inflammatory cells, but it can also be directly released from cancer cells^8^,^19^,^21^. There is increasing interest in the therapeutic potential of purinergic signaling for the treatment of cancer. The anti-neoplastic activity of ATP was first shown by Rapaport and collegues and more recent studies revealed an anti-tumor activity of extracellular nucleotides different cancer types^22^,^23^. The functional significance of purinergic signaling has been investigated on cancer cells but not on tumor endothelium^8^,^23^,^24^. P2 receptors are highly expressed by virtually all tumors and positive or negative modulation of P2 subtypes promotes cancer cell death or growth inhibition. In particular, altered P2X7R expression and function could play a role in tumor progression^25^. However, whether P2X7R exerts a promoting or suppressive role on tumor growth is still a controversial issue and its underlying mechanism remains unknown^26^,^27^. Interestingly, ATP-P2X7R pathway is clinically relevant for anthracycline treated breast cancer patients^28^. P2X7R could also act as proangiogenic mediators through the induction of VEGF release from tumor cells and human blood-derived monocytes^29^,^30^. Recently, P2X7R was proposed as a non-redundant host factor in *in vivo* anticancer response^31^.

As aforementioned, the direct role of purinergic signaling in tumor vessels and vascular endothelium is unknown.

Here we suggest that strong purinergic stimulation inhibits tumor-derived EC migration and enhances pericyte attraction, potentially leading to vessel normalization. These events are mediated by the interplay between calcium and cAMP signaling mainly triggered by P2X7 and P2Y11 receptors.

This work provides a novel mechanistic insight and unveils a new and potentially attractive role for purinergic signalling as stabilizing and normalizing agent for tumor vasculature, which could be used to develop novel anti-angiogenic therapeutic strategies.

## Results

### High ATP concentrations selectively inhibit migration and tubulogenesis of tumor-derived endothelial cells from human breast carcinoma, BTEC

Extracellular ATP was not toxic in the range considered (1 μM–1 mM for 24 h) for both normal EC (HMEC) and tumor-derived EC from breast carcinoma, BTEC (Supplementary Figure A), while a mitogenic activity was detectable upon 48 hrs of treatment on HMEC but not on BTEC (Supplementary Figure B).

**Figure 1:**
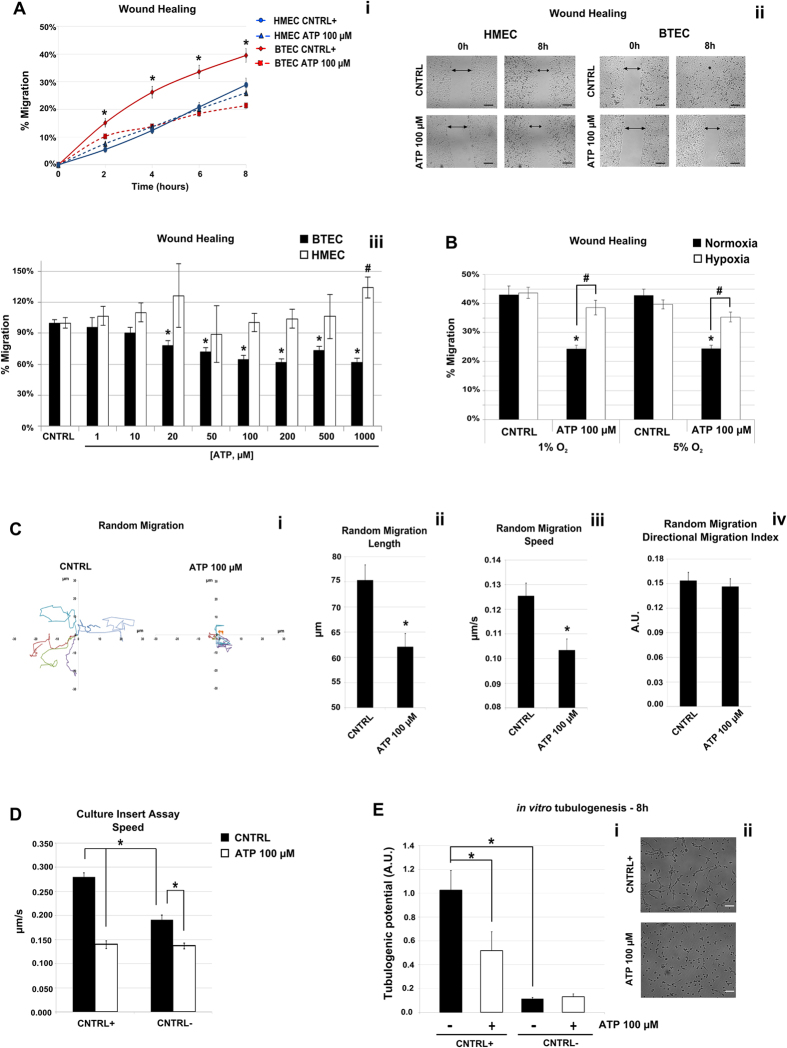
High concentrations of ATP inhibit BTEC migration. (**A,i**) Representative time course of a wound healing experiment on HMEC (blue traces) and BTEC (red traces) upon stimulation with ATP 100 μM (dotted lines) compared to positive control (solid lines). Data are expressed as mean ± S.E.M. Wilcoxon test: *p < 0.0001 vs. BTEC ATP 100 μM. (**A,ii**) Representative images relative to the previous figure; scale bar = 200 μm. (**A,iii**) Percentage of migration at 8 hrs in BTEC (black bars) and HMEC (white bars) stimulated with different ATP concentrations (1–1000 μM) in positive control (CNTRL) in at least three independent experiments for each condition. Data are normalized to the corresponding CNTRL and are expressed as mean ± S.E.M Wilcoxon test: *p < 0.0001 vs. CNTRL in BTEC; ^#^p < 0.001 vs. CNTRL in HMEC. (**B**) Representative wound healing experiment performed in BTEC under normoxic (21% O_2_) and hypoxic (1% or 5% O_2_) conditions. Percentage of migration at 8 hrs in normoxia (black bars) or hypoxia (white bars) in control and ATP 100 μM-stimulated cells. Data are expressed as mean ± S.E.M. Wilcoxon test: *p < 0.001 vs. CNTRL; ^#^p < 0.001. (**C,i**) Representative single cell free migration is reported in the two plots. BTECs were treated or not with ATP 100 μM: each line represents the migration of a single cell followed for 10 hours. The data shown are from a single representative experiment out of three. (**C,ii–iii**) Total length, speed and directional migration index were evaluated from three independent experiments. Data are expressed as mean ± S.E.M. Wilcoxon test: *p < 0.001 vs. CNTRL. (**D**) Effect of ATP 100 μM on migration rate (evaluated as cell speed in μm/s) of confluent BTEC plated in a chamber of silicone culture inserts (IBIDI). See ‘Culture-Insert Assay’ in Material & Methods for specification. Data are expressed as mean ± S.E.M. Wilcoxon test: *p < 0.01. (**E,i–ii**) Representative of three independent experiments of *in vitro* tubulogenesis on BTEC treated (white bars) or not (black bars) with ATP 100 μM in positive (CNTRL+) and negative control (CNTRL−) at 8 hrs. Total length and tubule complexity were measured for each field. Data are expressed as mean ± S.E.M. Wilcoxon test: *p < 0.01. Scale bar = 100 μm.

The effect of ATP on EC migration was first evaluated by *in vitro* scratch-wound healing assay. Incubation with ATP (1 μM–1 mM) failed to induce any significant effect on HMEC migration up to 8 hrs (Fig. 1A,i–iii). In contrast, addition of ATP at concentrations higher than 20 μM drastically reduced migration of BTEC, starting from 2 hrs of treatment (Fig. 1A,i–iii). This activity was significantly prevented when BTEC were cultured in moderate or severe hypoxic conditions (respectively 5% or 1% O_2_; Fig. 1B): severe hypoxia promoted BTEC viability at 7 and 24 hrs (Supplementary Figure D). In order to elucidate the molecular mechanisms underlying the antimigratory activity selectively exerted on BTEC by high purinergic stimulation, all the following experiments were performed in normoxia upon incubation with 100 μM ATP.

BTEC motility was reduced also in random migration assay starting from sparse cells (see methods). In particular, path length and speed were drastically decreased by 100 μM ATP (Fig. 1C,i–iii), while directional migration index (persistence) resulted unaffected (Fig. 1C,iv). The same trend was observed in insert-based migration starting from confluent cells (see methods, Fig. 1D).

To further investigate the biological roles of purinergic stimulation in TEC, we studied vascular morphogenesis *in vitro* by 3D culture Matrigel tubulogenesis. Treatment with 100 μM ATP significantly hampered tube formation upon 8 hrs of incubation (Fig. 1E,i–ii).

### Purinergic antimigratory effect is associated with cytoskeleton remodeling

Since cell migration is strictly associated with cytoskeleton remodeling, we decided to explore the effects of purinergic stimulation on actin and focal adhesion distribution in BTEC and HMEC.

**Figure 2:**
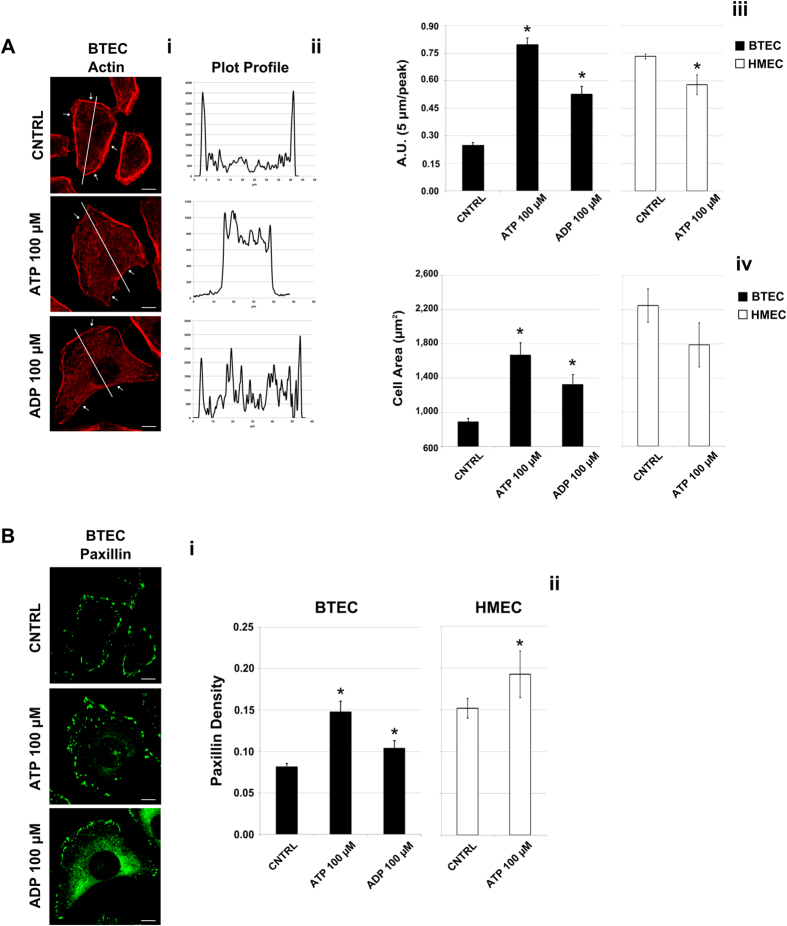
ATP 100 μM remodels BTEC cytoskeleton. (**A,i**) Actin staining with phalloidin in BTEC treated for 5′ with ATP 100 μM, ADP 100 μM and in control condition (CNRL); arrows indicate actin filaments distribution. Scale bar = 10 μm. (**A,ii**) Plot profile of the phalloidin fluorescence intensity along the major cell axis (white lines in the figures) and quantification of the cortical actin localization **(A,iii)** are reported for BTEC (black bars) and HMEC (white bars). Total area measured for each treatment is reported in the histograms **(A,iv).** Data obtained from three independent experiments are expressed as the mean ± S.E.M. Wilcoxon test: *p < 0.001 vs. corresponding CNTRL. (**B,i**) Representative images of focal adhesion staining with paxillin antibody in cells treated with ATP 100 μM, ADP 100 μM and in control condition are reported. Scale bar = 10 μm. Quantification of focal adhesion density is shown in the histogram (**B,ii**) for BTEC (black bars) and HMEC (white bars). Data from three independent experiments are expressed as mean ± S.E.M. Wilcoxon test: *p < 0.001 vs. corresponding CNTRL.

Immunofluorescence confocal microscopy revealed that acute treatment of BTEC with 100 μM ATP for 5′ leads to a significant rearrangement of both intracellular actin and focal adhesions (Fig. 2). In particular, the actin network is redistributed from a prevalent cortical to a more homogeneous pattern (Fig. 2A,i–iii) and focal adhesion density stained with paxillin was enhanced and more homogeneously distributed (Fig. 2B,i–ii). Moreover, 5′ stimulation with 100 μM ATP significantly increased EC area (Fig. 2A,iv), in accordance with a quiescent state of BTEC and the consequent reduction of the speed observed upon ATP treatment (see Fig. 1).

These events are selectively induced on BTEC, at least in part, since the same stimulation with ATP (100 μM, 5′) exerted a small but significant paxillin redistribution in HMEC (Fig. 2B,ii) but did not alter areas and intracellular actin distribution (Fig. 2A,iii–iv).

Interestingly, 100 μM ADP, a proinflammatory ATP catabolite, mimicked both actin and paxillin redistribution as well as the increase of BTEC area observed upon stimulation with 100 μM ATP (Fig. 2).

### Pharmacological and molecular evidence for a role of P2X7R and P2Y11R in ATP-mediated inhibition of TEC migration

In order to identify the purinergic receptors involved in the effects described above, we tested different agonists on BTEC migration: 100 μM ADP that binds to P2YR1/12/13, UTP acting on P2YR2/4/6, and 100 μM Adenosine (ADO) selective for P1R.

**Figure 3:**
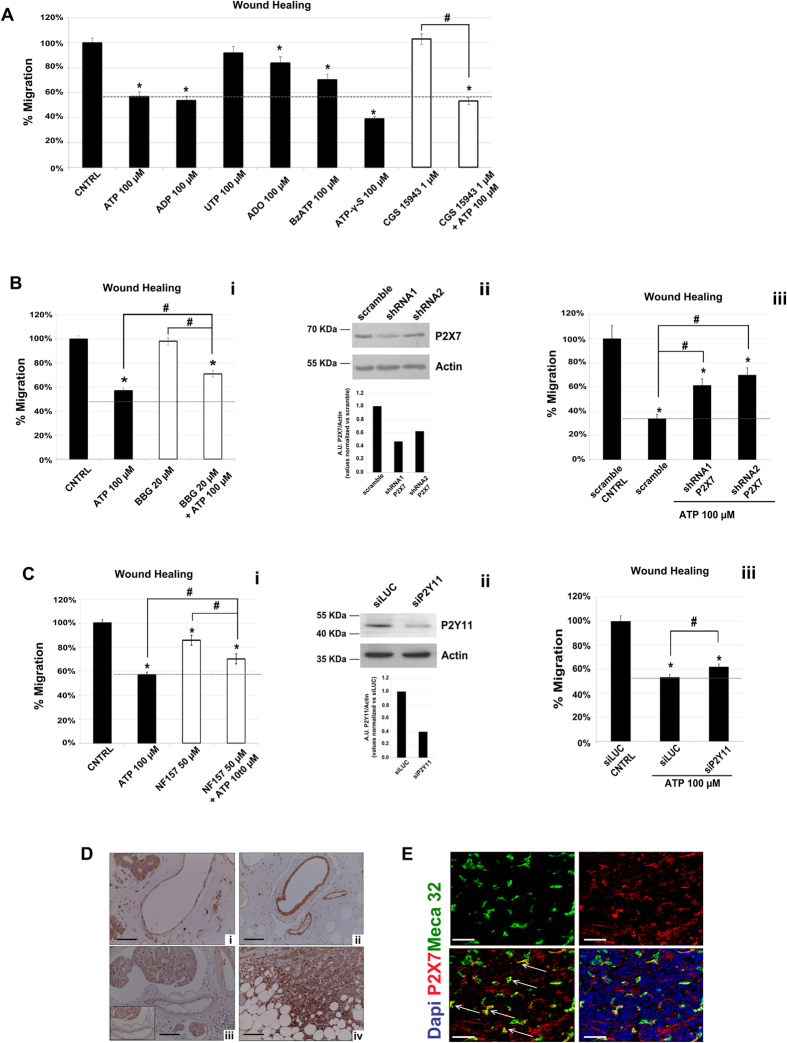
P2X7 and P2Y11 receptors are involved in BTEC-inhibitory effect induced by ATP 100 μM. **(A**) Wound healing at 8 hrs in BTEC treated with different molecules. Migration rate was normalized to positive control (CNTRL). Dotted line represents the ATP 100 μM-inhibitory effect. Data are expressed as mean ± S.E.M. Wilcoxon test: *p < 0.0001 vs. CNTRL; ^#^p < 0.001. (**B,i**) Wound healing experiments at 8 hrs in cell treated with the P2X7R inhibitor BBG 20 μM. Data are normalized to positive control and are expressed as mean ± S.E.M. Wilcoxon test: *p < 0.0001 vs. CNTRL; ^#^p < 0.001 (**B,ii**) Blot densitometry quantification after 72 hrs, of P2X7 expression in BTEC transfected with two specific shRNA. A generic scramble vector was used as control and to normalize data. Western blot image has been cropped (full-length blot is presented in Supplementary Figure D,i**–**ii) **(B,iii)** Wound healing experiments performed with transfected BTEC. Data from a representative experiment were normalized at 8 hrs to the scramble-CNTRL and expressed as mean ± S.E.M. Wilcoxon test: *p < 0.001 vs. scramble-CNTRL; ^#^p < 0.01. **(C,i**) Representative wound healing of BTEC preincubated with the P2Y11 inhibitor NF157 50 μM. Data were normalized to the CNTRL at 8 hrs and expressed as mean ± S.E.M. Wilcoxon test: *p < 0.0001 vs. CNTRL; ^#^p < 0.001 **(C,ii)** Western blot of BTEC transfected with siP2Y11 or relative control (siLUC). Blot densitometry quantification of P2Y11 expression normalized to siLUC control are shown as cropped image (full-length blot is presented in Supplementary Figure E,i**–**ii) **(C,iii**) Wound healing assay with IBIDI after 24 hrs from transfection. Data from a representative experiment were normalized at 8 hrs to siLUC control. Data are expressed as mean ± S.E.M. Wilcoxon test: *p < 0.001 vs. siLUC; ^#^p < 0.01. **(D,i–ii**) P2X7 Immunostaining in normal human breast tissue and in vessel structures (Magnification 10x). P2X7 immunostaining in endothelial cells near to neoplastic tissue (Magnification 10x and 20x) (**D,iii**) and in breast cancer cells or in inflammatory cells surrounding breast cancer tissue (Magnification 10x) (**D,iv**). Scale bar = 50 μm (**E**) P2X7 expression and localization assessed by confocal analysis of mammary tumors from 4T1-injected mice. Co-staining with Meca32 (green) revealed that P2X7 (red) was highly expressed in both vessels (arrows) and tumor cells in baseline conditions. Scale bar = 50 μm.

The inhibitory effect of 100 μM ATP on BTEC migration was reproduced by 100 μM ADP but not by 100 μM UTP, raising the possibility for an involvement of a subpopulation of P2YR as well as of P2XR (Fig. 3A).

In addition to ADP, 100 μM Adenosine (ADO), a potent endogenous agonist for P1 receptors, reduced BTEC migration, even if at lesser extent compared to ATP and ADP (Fig. 3A). However, 100 μM ATP-γ–S, a non hydrolysable form of ATP, nicely reproduces the inhibitory action of ATP, suggesting that the anti-migratory effect of ATP *in vitro* does not strictly requires its metabolism (Fig. 3A). Accordingly, the specific P1 receptor inhibitor CGS 15943 (1 μM) failed to restore cell migration inhibited by ATP (Fig. 3A).

Once excluded a significant role for P1R and many P2YR (UTP-sensitive), we decided to evaluate the involvement of P2XRs, particularly focusing on P2X7R for two reasons: i) a number of reports converge on P2X7R overexpression in cancer; ii) P2X7R displays a very low affinity for ATP, resulting selectively recruited by high ATP concentrations usually found in tumor microenvironment.

Notably, BTEC preincubation with 100 μM BzATP, an agonist preferentially acting on P2X7R and P2Y11R, drastically reduced cell migration, similarly to 100 μM ATP (Fig. 3A). The key role of P2X7R was confirmed by the partial recovery of migration in cells treated with 100 μM ATP in the presence of 20 μM BBG, a specific P2X7 antagonist (Fig. 3B,i).

Since BzATP recognizes both P2X7R and P2Y11R, we decided to confirm the pharmacological data described above by the molecular silencing of these receptors. We performed wound healing assays on P2X7R-shRNA and P2Y11R-siRNA silenced BTEC. P2X7R silencing after transfection was monitored by means of protein expression levels after 72h. The expression was reduced by about 53.2% for shRNA1 and 37.8% for shRNA2 (Fig. 3B,ii). P2X7 silencing was able to significantly rescue the ATP-dependent inhibition of cell migration (Fig. 3B,iii).

Given the aforementioned ability of BzATP to bind P2Y11R in addition to P2X7, we looked at its putative contribution. Pretreatment with 50 μM NF157, a specific P2Y11R inhibitor, partially rescued migration inhibited by ATP (Fig. 3C,i). A similar trend was observed in P2Y11R-silenced cells (Fig. 3C,ii–iii).

To evaluate whether our findings could be relevant for human cancers, we analyzed P2X7R expression in human breast specimens, in which both endothelial and epithelial cells show a positive P2X7R immunoreactions (Fig. 3D). Moreover, P2X7R expression was observed also in breast cancer cells, in inflammatory cells and in endothelial cells surrounding the neoplastic lesion (Fig. 3D). Similar pattern of P2X7R expression has been observed in a Orthotopic 4T1 mouse model of mammary tumor (see methods). As shown in Fig. 3E, immunofluorescence analysis revealed that P2X7R is expressed in tumor cells. More interestingly, co-staining of P2X7R with Meca32 clearly indicates its main expression in vessels inside the tumor mass (Fig. 3E).

### cAMP is involved in purinergic inhibition of BTEC migration and cytoskeleton remodeling

**Figure 4:**
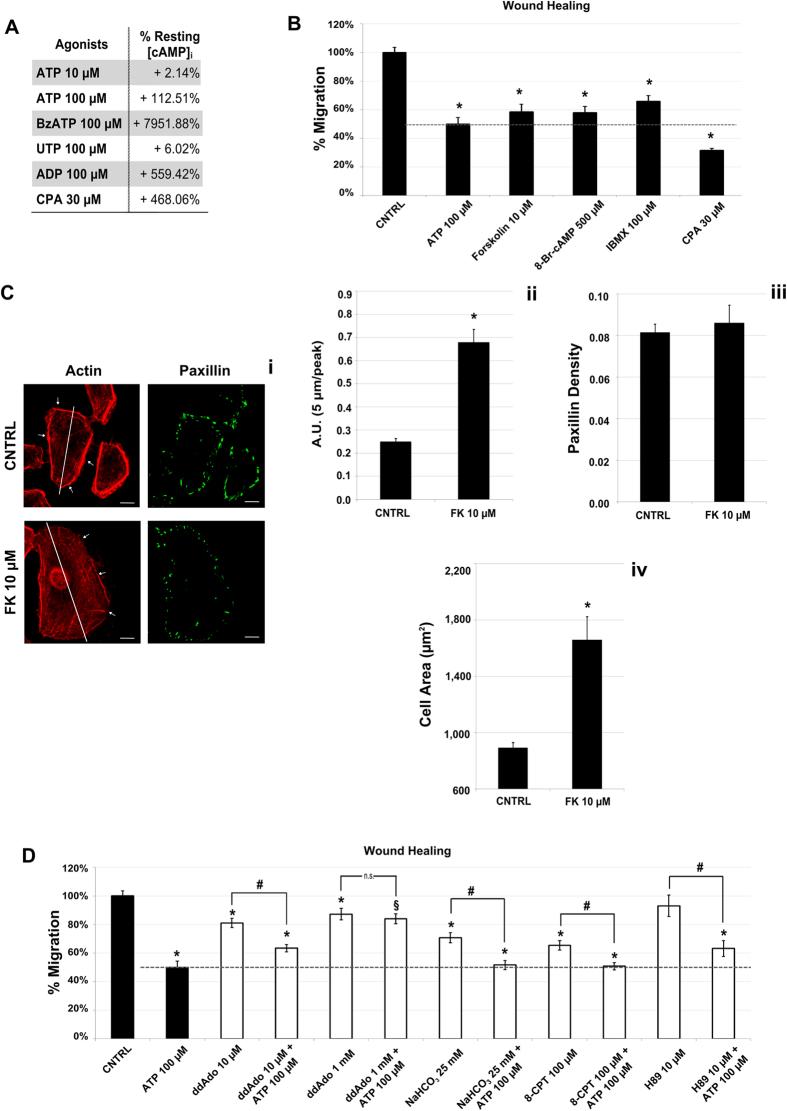
Reduction of BTEC migration is dependent on [cAMP]_i_ increase. (**A**) cAMP production upon stimulation with different purinergic agonists or CPA. ATP 100 μM and other antimigratory purinergic agonists induce a strong increase in intracellular cAMP level after 15′ of treatment, as shown in table A (data from at least three independent experiments are normalized to the control). (**B**) High cAMP levels are able to reduce BTEC migration in wound healing assay. Different inducers of intracellular cAMP increase were used: Forskolin 10 μM (FK), 8-Br-cAMP 500 μM and IBMX 100 μM. Data are normalized on positive CNTRL at 8 hrs and expressed as mean ± S.E.M. Wilcoxon test: *p < 0.001 vs. CNTRL; §p < 0.001 vs. ATP 100 μM; ^#^p < 0.001. (**C,i**) Actin and paxillin staining in cells treated with FK 10 μM or not (CNTRL) for 5′. Arrows in (C,i) indicate actin filaments distribution and phalloidin fluorescence intensity was evaluated along the major cell axis (white lines in the figures). Scale bar = 10 μm (**C,ii**) Quantification of the cortical actin localization. (**C,iii**) Paxillin density quantification upon FK treatment. (**C,iv**) Total area measured for each condition. Data obtained from three independent experiments are expressed as mean ± S.E.M. Wilcoxon test: *p < 0.001 vs. CNTRL. (**D)** Effect on BTEC migration (wound healing) of different adenylyl cyclase modulators (ddAdo, NaHCO_3_), EPAC-1 activator (8-CPT) and PKA inhibitor (H89). Dotted line represents ATP-induced inhibition. Data are normalized on positive CNTRL at 8 hrs and expressed as mean ± S.E.M. Wilcoxon test: *p < 0.001 vs. CNTRL; §p < 0.001 vs. ATP 100 μM; ^#^p < 0.001.

Among the signaling pathways triggered by purinergic stimuli on endothelium, the release of cyclic Adenosine Monophosphate (cAMP) has long been known. Since cAMP inhibits migration of different cell types, including endothelium^32^,^33^,^34^, we decided to evaluate its putative involvement in ATP-dependent anti-migratory activity on BTEC. Exposure to 100 μM ATP for 30′ increased cAMP levels (Fig. 4A) and an even stronger effect was induced by 100 μM ADP or 100 μM BzATP, showing that both P2YR and P2X7R concur to cAMP release. On the contrary, a lower ATP concentration (10 μM) and 100 μM UTP, both conditions that revealed unable to affect BTEC migration, resulted ineffective on cAMP levels (Fig. 4A). Interestingly, cAMP increase was also observed upon cell incubation with 30 μM CPA, that promotes calcium release from intracellular stores, suggesting that at least a component of cAMP elevation is calcium-dependent (Fig. 4A).

Accordingly, three compounds broadly used to promote cAMP release via different mechanisms (10 μM Forskolin, 100 μM IBMX, 500 μM 8-Br-cAMP) reproduced the inhibitory effect of ATP on cell migration (Fig. 4B). In the same way, CPA 30 μM drastically reduced BTEC migration.

In addition, preincubation of BTEC with Forskolin (10 μM, 5′) mimicked the remodeling of actin and area increase previously shown for 100 μM ATP, but did not alter paxillin distribution (Fig. 4C,i–iv). Selective isoforms of Adenylyl Cyclase (AC) are recruited: indeed, preincubation with high concentrations of AC inhibitor 2′,5′-dideoxyadenosine (ddAdo) (1 mM, 15′), selectively acting on soluble and calcium-activated AC10 isoform, led to a significant rescue from ATP-mediated antimigratory effect (Fig. 4D), while lower ddAdo concentration (10 μM), targeting AC5, decreased constitutive BTEC migration but failed to rescue from ATP-dependent anti-migratory activity (Fig. 4D). Moreover, cell preincubation with 25 mM NaHCO_3_, a selective activator of AC10, resulted anti-migratory, strengthening the hypothesis that this isoform could be a key component of purinergic signaling (Fig. 4D).

Finally, we looked at the signaling downstream to cAMP. Incubation with 100 μM 8-pCPT-2′-O-Me-cAMP, a selective activator of EPAC-1, a downstream target for cAMP, nicely reproduced the activity of ATP, and an additive effect was observed co-treating cells with both 8-pCPT-2′-O-Me-cAMP and 100 μM ATP (Fig. 4D). On the other hand, H89 (10 μM, 15′), a cell-permeable, non-selective, reversible and ATP-competitive inhibitor of PKA, was ineffective (Fig. 4D).

### High ATP concentrations ‘normalize’ BTEC *in vitro*

Based on the anti-angiogenic effects of 100 μM ATP on TEC *in vitro*, we evaluated the putative role of purinergic stimulation on tumor-derived endothelial permeability and vascular stabilization.

**Figure 5:**
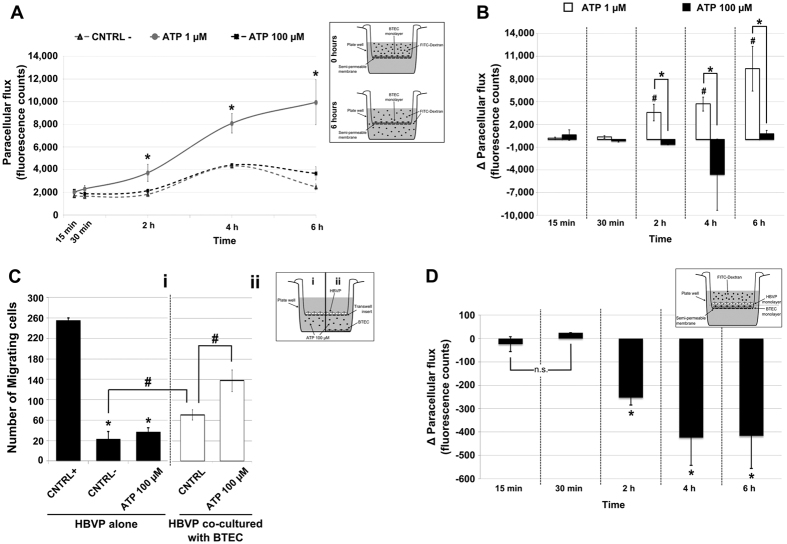
ATP 100 μM ‘normalizes’ BTEC *in vitro*. (**A**) Paracellular flux through a BTEC confluent monolayer was evaluated with 70 kDa Dextran-FITC (see the inset of Figure A and material and methods for more details). A representative time course analysis (6 hrs) of a single experiment. (**B**) Quantification of paracellular flux as the difference between ATP-treatment (ATP 1 μM in white bars and ATP 100 μM in black bars) and negative control for each time. Data obtained from three independent experiments are expressed as mean ± S.E.M. T-test ^#^p < 0.05 vs. fluorescence at 15′ and 30′; *p < 0.05. (**C,i**) Human brain vascular pericyte (HBVP) transwell migration after treatment in the lower chamber with ATP 100 μM, positive (CNTRL+) and negative (CNTRL-) controls. In the histogram (black bars) is reported the number of migrating cells. (**C,ii**) HBVP migration plating BTEC in the bottom chamber and treating them or not with ATP 100 μM for 3hrs (white bars) before transwell migration assay. See inset for more specification. Data show a representative experiment and are expressed as mean ± S.E.M. T-test *p < 0.01 vs. CNTRL+; ^#^p < 0.05. (**D**) Dextran-FITC paracellular flux evaluated seeding confluent HBVP over the BTEC monolayer. Histograms report the mean difference of three independent experiments between the flux obtained with BTEC alone and the co-plated BTEC-HBVP cell lines. In both conditions cells were treated with Histamine 100 μM. See inset for more specification. Data are expressed as the mean ± S.E.M. T-test: *p < 0.05 vs. fluorescence at 15′ and 30′.

Since purinergic signaling is usually associated to an increase in vascular permeability, we performed *‘in vitro’* permeability assays by the use of fluorescent tracers. BTEC permeability was significantly enhanced upon 6 hours incubation with 1 μM ATP but, notably, it resulted completely unaffected upon treatment with 100 μM ATP (Fig. 5A,B).

Finally, we tested the ability of BTEC to recruit pericytes, the major actors involved in vessel stabilization *in vivo.* ATP alone did not exert any direct effect on pericyte migration, as revealed by transwell assays (Fig. 5C,i). Conversely, the presence of BTEC was chemoattractant for pericytes and, more importantly, the effect was potentiated when cells were pretreated with 100 μM ATP (3 hours) (Fig. 5C,ii). This event can be viewed as an early step of pro-normalizing effect promoted by high ATP concentrations: indeed, co-culturing BTEC with pericytes significantly reduced permeability when compared with the BTEC cultured alone (Fig. 5D).

## Discussion

In the present study we evaluated for the first time the effects of extracellular ATP and other nucleotides on vascular remodeling of healthy and altered endothelium *in vitro,* respectively by the use of human microvascular EC types from normal dermal vasculature (HMEC), and breast carcinoma (BTEC).

We chose ATP concentrations ranging from the physiological levels to higher doses associated with inflammatory or tumoral environment (1 μM–1 mM). Indeed, recent luciferase-based sensors for ATP unveiled that ATP in healthy tissues is below 1–5 μM, reaching hundreds of μM in tumor interstitium^21^. Interestingly, all the concentrations were mitogenic for normal EC (HMEC), but ineffective on the tumor-derived type (BTEC), in agreement with the peculiar features of TECs reported previously by our group and others^35^.

The most relevant difference between TEC and normal endothelium was observed on migration assays. Higher ATP concentrations (>20 μM), usually found in tumor microenvironment, exerted a robust anti-migratory activity on BTEC, but resulted ineffective on HMEC. This unexpected evidence was confirmed by different independent approaches (i.e. scratch wound healing, culture-insert assays, random migration) and it is associated to the cytoskeleton rearrangements typically observed when cell migration is altered.

Purinergic-dependent anti-migratory effect has been previously reported for human acute myeloblastic leukemia cells and human dendritic cell types through the P2Y11R signaling^36^,^37^, but this is the first evidence on vascular endothelium.

**Figure 6:**
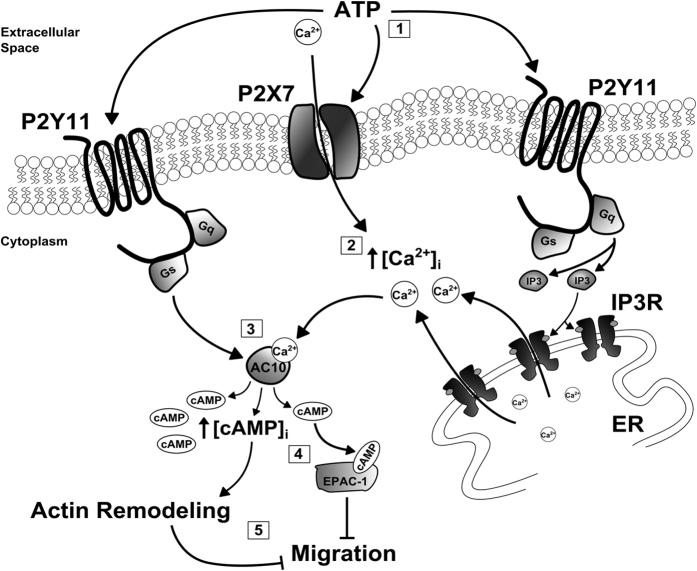
Schematic representation of the molecular mechanisms underlying the inhibitory effect on BTEC migration. Extracellular ATP recruits P2Y11 and P2X7 purinergic receptors (1). P2Y11 activation leads to InsP3 production through Gq protein and PLCβ. Calcium influx through P2X7 receptor channels, together with calcium release from the ER, further increase [Ca^2+^]_i_ (2). Direct activation of adenylyl cyclase 10 (AC10) through Gs protein upon P2Y11 stimulation, or calcium-dependent AC engagement (3), increase intracellular cAMP levels leading to cytoskeleton remodeling. High cAMP concentrations recruit EPAC-1(4) which mediates the inhibitory effect on migration (5).

An important section of the paper is focused on the molecular mechanism underlying the anti-migratory effect promoted by ATP in TEC (see scheme in Fig. 6 for an overall view of the main conclusions).

The inhibition of BTEC migration by high ATP levels was mimicked by ADP and partly by BzATP, but not by UTP, strongly suggesting the involvement of a subpopulation of P2YRs as well as of P2X7R. Adenosine reduced BTEC migration, even if to a lesser extent than ATP. Since extracellular ATP can be hydrolyzed to Adenosine through the involvement of ectonucleotidases, we tested the possibility that it could contribute to the inhibition of BTEC migration induced by ATP. The ability of ATP-γ–S to reproduce ATP-mediated inhibition of migration points to a minor role of ADP and Adenosine *in vitro*, but we cannot exclude their involvement *in vivo*. This observation agrees with the lack of rescue by treatment with 1 μM CGS 15943, a selective P1 receptor antagonist.

We provide a number of independent evidences (pharmacology and molecular silencing) on the critical involvement of P2Y11 and P2X7 receptors, respectively a G-protein coupled and an ion channel receptor. In particular, the low affinity of P2X7R for ATP provides a nice mechanistic correlation with the evidence that only high concentrations of ATP inhibit BTEC migration. Notably, here we show for the first time that P2X7R is expressed *in vivo* in vessels of murine and human breast tumors. The antimigratory role of P2X7R on BTEC offers an intriguing explanation for its complex (and somehow contradictory) regulatory role in cancer progression and should be integrated with its overexpression observed in cancer cells^25^. Nonetheless, our data opens a more complex scenario in which P2X7R is not the only regulatory actor for BTEC migration, as revealed by the contribution of P2Y11R.

Among the intracellular messengers that negatively regulate cell migration, cAMP has been well described^32^,^33^,^34^,^38^. Moreover, its production inhibits vascular permeability of EC monolayers^38^. A number of purinergic receptors are coupled with adenylyl cyclases (AC) and cAMP release. Notably, both P2Y11R and P2X7R activate AC directly or indirectly, via calcium-dependent or G-protein coupled AC isoforms^23^. cAMP reduces proliferation and migration on endothelial cells and, accordingly, cyclic nucleotide phosphodiesterases (PDE) regulate endothelial migration and angiogenesis^39^.

Here we show that high ATP raises intracellular cAMP and that different cAMP-releasing compounds inhibit BTEC migration. In agreement with these data, we observed a significant rescue from ATP-mediated anti-migratory effect by AC inhibition with ddAdo. Some AC isoforms are calcium-dependent^40^ and the ability of CPA to promote cAMP release suggests that they are expressed and functional in BTEC. Moreover, besides cAMP signaling, most purinergic receptors trigger cytosolic Ca^2+^ increases in endothelium: G-protein coupled P2YRs usually release Ca^2+^ from intracellular stores, while P2XRs are ion channels that promote Ca^2+^ entry from the extracellular medium. We took advantage from the concentration-dependent targeting of AC-inhibitor ddAdo, in addition with the sensitivity to bicarbonate, to demonstrate a differential role for endothelial AC10 and AC5 isoforms in response to purinergic stimula in tumor microenvironment. These observations could be a valuable background for future work specifically devoted to the molecular mechanisms and functions of the interplay between calcium and cAMP signaling in tumor endothelium.

Interestingly, BTEC migration was significantly decreased by activation of EPAC-1, a downstream target or cAMP, accordingly with the ability of EPAC-1 to inhibit migration reported on different cell types, including cancer cells^32^,^33^. Moreover, prolonged stimulation of Rap1 either by EPAC activation following treatment with 8-pCPT or by expression of constitutively activated Rap1 A63E (cRap1) inhibits chemotaxis and angiogenesis in primary HMEC^41^. It is worth noting that cAMP/EPAC-1/Rap-1 pathway is involved in HAEC, HPAEC and HUVEC barrier stabilization^38^,^42^,^43^. This event inhibits the permeability independently of PKA by cytoskeleton rearrangement and augmentation of VE-cadherin-mediated cell-cell adhesion in HUVEC and HAEC^44^. Accordingly, in our hands PKA inhibition by H89 failed to rescue ATP-induced anti-migratory activity.

In the last part of this work we asked whether purinergic stimulation could be associated with vascular normalization. Indeed, following Jain’s original proposal, anti-angiogenic treatments can remodel tumor vasculature leading to permeability reduction, the following decrease of the interstitial pressure, and the increase of pericyte coverage with an overall stabilization of the abnormal tumor-associated vasculature. Therefore, tumor blood flow becomes more efficient in delivering cytotoxic drugs and oxygen for radiotherapy^45^,^46^,^47^.

ATP is commonly viewed as a proinflammatory agent that promotes vascular permeability^8^,^48^. Here we show that 1 μM ATP (ineffective on cell migration) increases BTEC permeability, whereas treatment with 100 μM ATP is ineffective: instead, it enhances BTEC-mediated pericyte migration, with the consequent coverage by pericytes that significantly decreases endothelial permeability. Since ATP is unable to attract pericytes in the absence of BTEC, the coverage is likely due to an indirect paracrine cross-talk between the two cell types, that should deserve future investigations. In particular, a number of soluble endogenous pro-normalizing factors have been described, including PDGF, Angiopoietin 1, TGFβ and Semaphorin 3A^45^: a detailed investigation of the their involvement, or additional pathways, will help to unveil a novel molecular machinery for tumor vessel normalization.

Taken together, data presented here lead us to conclude that high ATP concentrations found in tumor microenvironment act on mature TEC as an endogenous stabilizing and pro-normalizing factor.

Purinergic effectiveness could be limited *in vivo* by at least two environmental factors. Firstly, here we show that hypoxia, usually found in deep tumor mass, significantly prevents the anti-angiogenic activity of high ATP. This intriguing observation could be explained by an alteration of P2X7R expression, targeting or functionality. Azimi *et al*. recently assessed ATP-mediated calcium signaling in a model of hypoxia-induced epithelial-mesenchimal transition (EMT) in MDA-MB-468 breast cancer cells: 1% O_2_ treatment was associated with a significant reduction in the cell sensitivity to ATP (EC50 of 0.5 mM for normoxic cells versus EC50 of 5.8 mM for hypoxic cells). mRNA levels for P2X4, P2X5, P2X7, P2Y1 and P2Y11 decreased with hypoxia, whereas P2Y6 mRNA increased^49^. This could be the case for BTEC and future evaluation of transcript and protein levels should be performed in order to test this hypothesis.

Another constraining factor to the potential antiangiogenic power of P2X7, that is shown here to be expressed in tumor vessels *in vivo* and functionally required for vascular stabilization *in vitro,* may be related to an peculiar biophysical feature of this ion channel: its sensitivity to extracellular acidosis, a condition occurring at hypoxic sites of inflammation and cancer^50^. P2X receptors are functionally regulated by acidic pH with differential effects depending on the receptor subtypes: extracellular pH decrease facilitates P2X2 and P2X2/3 receptors but inhibits P2X1, P2X3, P2X4 and P2X7 receptors^51^. Site-directed mutagenesis studies show the differential role of extracellular histidines in copper, zinc, magnesium and proton modulation of the P2X7 purinergic receptor^52^. Lys110 and particularly Asp197 are involved in the functional inhibition of rat P2X7 receptor by acidic pH^53^.

In principle, P2X7R defective expression and function due to the peculiar tumor microenvironment are not mutually exclusive and could both contribute to the actual role of purinergic agonists on tumor vessels.

## Material and Methods

### Cell Cultures and Transfection

Breast tumor-derived endothelial cells (BTEC) from human breast lobular-infiltrating carcinoma biopsy were isolated and provided by the laboratory of Professor Benedetta Bussolati (Department of Internal Medicine, Molecular Biotechnology Center and Research in Experimental Medicine Center, University of Torino, Italy) and periodically characterized for the expression of endothelial markers. BTEC were grown in EndoGRO-MV-VEGF Complete Media Kit composed of EndoGRO Basal Medium and EndoGRO-MV-VEGF Supplement Kit (Merck Millipore). EndoGRO Basal Medium is a low serum culture media for human microvascular endothelial cells, supplemented with a kit containing rhVEGF (5 ng/ml), rhEGF (5 ng/ml), rhFGF (5 ng/ml), rhIGF-1 (15 ng/ml), l-glutamine (10 mM), hydrocortisone hemisuccinate (1.0 μg/ml), heparin sulfate (0.75 U/ml), ascorbic acid (50 μg/ml), fetal bovine serum FCS 5%. Human microvascular endothelial cells (HMEC), are dermal-derived cells (HMEC) purchased from Lonza. HMECs were grown in EndoGRO-MV-VEGF Complete Media Kit, as well as BTEC.

Human Brain Vascular Pericytes (HBVP) were provided by Prof. Enrico Giraudo and Dr. Federica Maione (Laboratory of Transgenic Mouse Models, Candiolo Cancer Research Center, Italy). HBVP were grown in Pericyte Medium (PM - ScienCell) supplemented with 2% FCS and pericytes growth supplement.

All cell cultures were maintained in normoxic (37 °C, 21% O_2_ and 5% CO_2_) and, when needed, hypoxic (37 °C, 1% O_2_ or 5% O_2_ and 5% CO_2_) incubator (InVIVO_2_ 200 equipped with a Ruskinn Gas Mixer Q) using Falcon plates as supports (about 5000 cells/cm^2^) and were used at passage 3 to 15. Only for BTEC, cells were cultured on a 1% gelatin layer.

Some experiments were performed using DMEM, Dulbecco’s modified eagle medium (Sigma-Aldrich Corporate, MO, USA), with 4500 mg/L glucose, 15 mM HEPES and sodium bicarbonate. DMEM was supplemented with 2% l-glutamine, 0.5% gentamicin, and 0, 2, or 10% fetal calf serum (FCS, Sigma).

BTEC cells were transfected with 2 μg shP2X7–1, shP2X7–2 (kindly provided by Prof. Francesco Di Virgilio, Department of Morphology, Surgery and Experimental Medicine, University of Ferrara, Italy)^25^ or a generic Scramble plasmid using the Amaxa Basic Nucleofector Kit for mammalian ECs according to the instructions of the manufacturer using the M-003 program (Lonza).

Transfection with siRNA was performed with Gene Pulser Xcell Electroporation Systems (Bio-Rad) according to the instructions of the manufacture. The control siRNA (pGL2 Luciferase) and the P2Y11-targeting siRNA (5′-UAUGUCUGCAAAGCUCGGGCAGCGG-3′) were obtained from Eurogentec S.A. Overall, 1·10^6^ BTEC were transfected.

### Chemicals

BBG purchased from Santa Cruz Biotechnology. ATP, ADP, UTP, ADO, CPA, H89, IBMX, BzATP, 8-Br-cAMP, Fluorescein isothiocyanate–dextran (average mol. wt. 70,000 Da) purchased from Sigma; 8-(4-Chlorophenylthio)-2′- O- methyladenosine-3′, 5′-cyclic monophosphate (8-pCPT-2′-O-Me-cAMP, 8CPT) purchased from BioLog; Forskolin (FK), CGS 15943 and NF157 purchased from Tocris Bioscience. 2′,5′-Dideoxyadenosine (ddAdo) purchased from Enzo Life Sciences. See Appendix for a more detailed list, with the concentrations used and molecular implications.

### Proliferation Assay

Cells were plated (500 cells/well) in DMEM, Dulbecco’s modified eagle medium (Sigma-Aldrich Corporate, MO, USA), supplemented with 10% FCS (fetal calf serum) and grown on 96-well culture plates (coated with 1% gelatin for BTECs). After 24 hours, cells were starved in DMEM containing 0% FCS (for BTEC) or 2% FCS (for HMEC). Fresh DMEM with or without agents to be tested was added (5 wells/condition) 24 hours later. Treatments were dissolved in DMEM 0% FCS (for BTEC) or 2% FCS (for HMEC). DMEM 10% FCS was used as positive control, whereas DMEM 0% or 2% FCS served as negative controls. After 48 hours of treatment, cells were colored using the CellTiter 96 AQueous One Solution cell proliferation assay (Promega, USA) and absorbance was recorded at 490 nm in a microplate reader (model 550, Bio-Rad) after 3 hours of incubation.

### Cytotoxicity Assay

Cells were plated (5000 cells/well) in DMEM 10% FCS plus treatments on 96-well culture plates (5 wells/condition). DMEM 10% FCS was used as positive control. After 24 hours of treatment, cells were colored using the CellTiter 96 AQueous One Solution cell proliferation assay as mentioned above.

### Migration assays

*Scratch Wound Healing Assay.* BTEC and HMEC were plated (25000 cells/cm^2^) using EndoGRO 5% FCS on 24-well culture plates coated with 1% gelatin. Cells were maintained in incubator until confluence was reached. Cell monolayers were starved for 6 hours in DMEM 0% FCS for both cell types. Motility assay was performed by generating a wound in the confluent cellular monolayers with a P10-pipette tip. Floating cells were removed by wash in PBS solution, and monolayers were treated with the different experimental conditions (in duplicate). EndoGRO 5% FCS was used as positive control, whereas DMEM 0% FCS (for BTEC) or 2% FCS (for HMEC) served as negative controls.

Wound healing experiments with P2Y11-transfected BTEC were performed plating cells in IBIDI culture inserts in both chambers. Cells were starved in DMEM0% and then IBIDI inserts were removed and treatments were added. This approach was necessary because the strongest reduction of P2Y11R expression was observed already after 24 hours, and these inserts allow to rapidly reach a confluent cell monolayer.

Experiments were performed using a Nikon Eclipse Ti (Nikon Corporation, Tokio, Japan) inverted microscope equipped with a A.S.I. MS-2000 stage and a OkoLab incubator (to keep cells at 37 °C and 5% CO2. Images were acquired at 2h time intervals using a Nikon Plan 4X/0.10 objective and a CCD camera. MetaMorph software (Molecular Devices, Sunnyvale, CA, USA) was used to acquire images for 5 time points.

#### Random Migration Assay

BTEC were plated (4000 cells/cm^2^) in EndoGRO 5% FCS on 12-well culture plates coated with 1% gelatin. The following day cells were starved for 6 hours in DMEM 0%. Cells were then washed with PBS solution and treatments were added in duplicate. EndoGRO 5% FCS was used as positive control, whereas DMEM 0% FCS was the negative control. Experiments were performed using the same set-up described above but using a Nikon Plan 10X/0.10 objective. Images were acquired for 10 hours every 15 minutes using MetaMorph software.

#### Culture-Insert Assay

BTEC were plated in EndoGRO 5% FCS on 12-well culture plates using silicone culture inserts (IBIDI GmbH, Planegg, Germany). Each insert had two 70 μl chambers, one of which was left empty while the other was used to plate cells (3–5 ∙ 10^5^ cells/well). Cells were maintained in incubator until confluence was reached. Cell monolayers were starved 6 hours in DMEM 0% FCS and subsequently the inserts were removed. Cells were then washed with PBS solution and treated. Experiments were performed in duplicate. EndoGRO 5% FCS was used as positive control, whereas DMEM 0% FCS was the negative control. Experiments were performed using the same set-up described for the wound healing assays with a Nikon Plan 15X/0.10 objective. Images were acquired for 6 hours every 10 minutes using MetaMorph software. BTEC transfected with siRNA were plated in wells and images were acquired for 8 hours (photos every 2 hours), using a 4X/0.10 objective.

#### Transwell Migration Assay

BTEC were plated (4000 cells/cm^2^) in EndroGRO 5% FCS on 24-well culture plates coated with 1% gelatin. The following day cells were treated for 3 hours with ATP 100 μM dissolved in DMEM 0% (except for the control). Then HBVP were added in Transwell Inserts with a 8 μm pore size PET membrane (BD Falcon) coated with Fibronectin (1 μg/ml). Transwell Inserts were then placed either in the 24-well culture plates over stimulated BTEC, or in wells without cells but containing DMEM 0% (CNTRL-), DMEM 10% FCS (CNTRL+) or DMEM 0% + ATP 100 μM. HBVP were let to migrate for 4 hours and at then Transwell Inserts were fixed with 4% Paraformaldehyde and stained with DAPI. Non-migrated cells were removed using a cotton swab. Total migrated cells (nuclei) were counted using a fluorescence microscope.

### *In vitro* Angiogenesis Assay

*In vitro* formation of ‘capillary-like’ structures was studied on growth factor-reduced Matrigel (BD Bioscience, NJ, USA). BTEC were seeded (35000 cells/well) onto Matrigel-coated 24-well plates in growth medium containing treatments (in duplicate). EndoGRO 5% FCS was used as positive control and DMEM 10% FCS as negative control. Cell organization into Matrigel was periodically observed with the same set-up as the one used for wound healing and one-side migration assays using a Nikon Plan 10X/0.10 objective. Images were acquired at 2h time intervals (9 time points) using MetaMorph software.

### Western blot analysis

BTEC transfected with siRNA for P2Y11/pGL2-Luciferase or shRNA for P2X7/Scramble were grown in EndoGRO 5% FCS in Petri dishes for 24 and 72 hours respectively. Protein were extracted, fractionated by SDS-PAGE and transferred onto a PVDF Transfer Membrane (Thermo scientific). The membrane was incubated with a rabbit polyclonal Ab directed against the human P2X7 receptor or P2Y11 receptor (Genetex) overnight at 4 °C, or 1h at RT for Actin. After washing, the membrane was incubated with HRP conjugated goat anti-rabbit Ab (Santa Cruz Biotechnology) and visualized using the ECL system (PerkinElmer) followed by autoradiography.

### Paracellular tracer flux assay

BTEC were seeded on 6.5-mm diameter Cell Culture Inserts contain PET membrane with 0.4 μm pore size (BD Falcon), cultured for 48 h in complete culture medium and assayed for permeability to fluorescein isothiocyanate (FITC)-dextran (70 kDa) (Sigma). When cells were confluent, 1 mg/ml of FITC-dextran was added to the medium of the insert apical compartment. At 15 min, 30 min, 2 h, 4 h and 6 h, aliquots of the medium ware collected from the basal compartment and the paracellular flux was measured using the fluorometer GloMax-Multi Detection System (Promega). EndoGRO with ATP 1μM was used as a positive control, while EndoGRO basal medium was used as negative control and to dissolve treatments. In experiments performed with human pericytes, BTEC were first plated in 0.4 μm pore size insert for 24 h in order to reach the confluence. Then HBVP were plated over the BTEC monolayer and cultured to complete confluence. Afterwards experiments were performed as described above and cells were treated with EndoGRO with Histamine 100 μM.

### cAMP measurements

cAMP levels were accessed using Screen Quest Colorimetric ELISA cAMP Assay Kit (AAT Bioquest), in accordance with the manufacturer’s instructions. Briefly, BTEC were seeded in a 96-well culture plates (30 · 10^4^ cells/cm^2^) and cultured overnight in complete medium. The next day, cells were incubated for 30 minutes with treatments dissolved in EndoGRO 5% FCS (CNTRL). Then cells were lysed and transferred into the anti-cAMP coated 96-well plate. HRP-cAMP conjugate solution and, after washing, Amplite Green were added. Absorbance was measured after 3 hours at 405 nm in a microplate reader.

### Immunofluorescence and confocal microscopy

BTEC and HMEC were plated on 12 mm glass dishes (13000 cells/cm^2^). The next day cells were incubated for 5 minutes with treatments dissolved in EndoGRO 5% FCS (CNTRL). Cells were then fixed with 4% Paraformaldehyde and permeabilized using Triton 0.1%. Staining of actin was achieved using the Rhodamine-labeled Phalloidin (Invitrogen). Staining of paxillin was achieved using a primary antibody (Millipore) and Alexa Fluor-488 Goat Anti-Rabbit IgG (H+L) antibody. Images were collected using a Leica TCS SP2 AOBS confocal microscope and analyzed using ImageJ software. In each field, we chose the stress fiber focal plane and analyzed the field with a plot profile of the major cell axis.

### Immunohistochemistry on human breast carcinoma biopsies

Immunohistochemistry staining for P2X7 (Rabbit Polyclonal antibody, GeneTex) was performed using Ventana Benchmark XT automated slide preparation system. Tissue sections (2–3 μm thickness) were deparaffinized (EZ-Prep, Ventana Medical Systems, at 75 °C) followed by antigen-retrieval (Cell Conditioning 1, Ventana Medical Systems, 30′ at 95–100 °C). Primary Antibody was incubated at room temperature for 32′, at 1:500 dilutions. Antibody staining was developed using the UltraView Universal DAB detection system (Ventana Medical Systems), and accompanied by hematoxylin counterstained.

The study was approved by the ethic institutional review board for “Biobanking and use of human tissue for experimental studies” of the Pathology Services of the ‘Azienda Ospedaliera Città della Salute e della Scienza di Torino’. Informed consent was obtained from all subjects prior to analysis. All methods were performed in accordance with the relevant guidelines and regulations.

### Immunohistochemistry in Orthotopic 4T1 Mouse model

Tumor cell lines were grown in standard medium supplemented with L-glutamine and 10% fetal bovine serum (Sigma). 4T1 spontaneous mouse mammary carcinoma cells were originally derived from a single spontaneous tumor arisen in a BALB/cfC3H mouse^54^.

In order to inoculate the cells within the fat pad of Balb/c mice we anesthetized the animals by means of 2.5% isoflurane anesthesia. After the inoculation mice were treated with analgesic (Rimadyl 2 mg/kg) and antibiotic (Baytril 2,5% 5 mg/kg) drugs. Mice were monitored throughout the experiments for complications eventually caused by the surgery. At the end of the experiments mice were anesthetized with isofluorane and euthanized by cervical dislocation.

#### Orthotopic 4T1 Mouse model

Six Balb/c mice were purchased from Charles River Laboratory (Calco, Milan, Italy) and 5  × 10^5^ 4T1 cells were injected subcutaneously into the flank or the mammary fat pad of anesthetized animals. Mice were typically euthanized 4 weeks after tumor cell injection, when the tumor size was more than 800/1000 mm^3^.

#### Tissue preparation and immunohistochemistry analysis

Immunofluorescence analysis was performed by using frozen tissues as previously described^55^. Briefly, cryostat sections (10 μm) were air-dried, fixed in zinc-fixative (6,05 g Tris, 0,35 g Ca(C_2_H_3_O_2_)2, 2,5 g Zn(C_2_H_3_O_2_)_2_,2,5 g ZnCl, 3,8 ml HCl 37%) for 10 minutes. Then tissues were blocked in 3% BSA and 5% donkey serum diluted in 1 × PBS and decorated with the Rabbit polyclonal anti-P2X7 (GeneTex) and the rat monoclonal anti-Panendothelial Cell antigen (clone Meca32, BD Pharmingen, USA) both diluted 1:100 in saturation solution. After rinse, the following secondary antibodies used were: anti-Rabbit Alexa Fluor-555 and anti-Rat Alexa Fluor-488. Finally, nuclei were counterstained with DAPI (Invitrogen). All immunofluorescence images were captured by using a Leica TCS SP2 AOBS confocal laser-scanning microscope (Leica Microsystems). All immune-localization experiments were performed on multiple tissue sections and included negative controls for determination of background staining, which was negligible. All *in vivo* experiments were carried out with the approval of the Italian Ministry of Health and the Ethics Committee of Candiolo Cancer Research Center (OPBA), in accordance with international laws and policies. Furthermore all the procedures followed the conditions established by the European Union.

### Data Analysis and Statistics

In proliferation and cytotoxicity assays, absorbance values obtained were analyzed with Excel software (Office, Microsoft). Absorbance values for each condition were normalized on the negative control, to assess whether there was stimulation or inhibition of cell growth (for proliferation assays) and cytotoxic effects (cytotoxicity assays).

Wound healing experiments and transfected cells migration with inserts were analyzed using Metamorph software. Cell migration was assessed by measuring the distance between the two sides of the wound at each time point. Obtained data were further analyzed using Excel in order to calculate the percentage of migration for each wound (difference of distances between cell fronts at two subsequent time points over distance at time 0) and the percentage mean value for each condition was then calculated. At least six fields for each condition were analyzed for every independent experiment. Where indicated, data were normalized to a control (100%) and relative migration was calculated, evaluating the propagation of errors for the S.E.M. of each treatment.

In Random migration and Culture-insert migration assays (with cells plated in one chamber), MTrackJ plugin of ImageJ software was used to calculate cells speed (μm/s) by measuring the distance between cell fronts of subsequent time points. For Random migration assay we considered the total length (μm), and we calculated the persistence as the ratio between the total length and D2S (the distance from the first start point of the track to the current point expressed in μm). Measurements were made for each time point and at least eight fields for each condition were analyzed for every experiment.

In *in vitro* angiogenesis assay, the number of nodes and tubule length were evaluated with ImageJ and normalized to maximum values. Their sum for each condition was used as an index for organization in ‘capillary-like’ structures in terms of arbitrary units (AU). At least ten fields for each condition were analyzed in each independent experiment.

For immunofluorescence image analysis ImageJ software was used. Quantifications of cortical actin were carried out using the fluorescence signals within the first 5 μm from the top of the cell, and calculating the ratio between the 5 μm fluorescence value and the peak amplitude within the 5 μm. Quantification of Paxillin density was calculated using the ImageJ function ‘Analyze Particles’ to count the number of focal adhesion spots, divided by the cell area.

In western blot, in order to quantify the differential protein expression, ratio between P2X7R or P2Y11R and actin expression was evaluated and then normalized on the corresponding control.

Paracellular flux was evaluated as the fluorescence difference between the treatment and the negative control (DMEM), putting together all the data of three independent experiments. In experiments performed with human pericytes plated over BTEC, we evaluated the fluorescence difference between the co-cultured inserts and BTEC only inserts.

Statistical analysis in most of the experiments was performed using the non-parametric Wilcoxon-Mann-Whitney test with Kaleidagraph software (Synergy Software). Where indicated, we performed Student’s t-test. Data considered significant showed a p-value (p) < 0.05. All experiments were carried out with, at least two, biological replicates for each experimental condition. All experiments were repeated at least three independent times.

## Additional Information

**How to cite this article**: Avanzato, D. *et al*. Activation of P2X7 and P2Y11 purinergic receptors inhibits migration and normalizes tumor-derived endothelial cells *via* cAMP signaling. *Sci. Rep.* **6**, 32602; doi: 10.1038/srep32602 (2016).

## Supplementary Material

Supplementary Information
