# Erratum: Activation of P2X7 and P2Y11 purinergic receptors inhibits migration and normalizes tumor-derived endothelial cells *via* cAMP signaling

**DOI:** 10.1038/srep35897

**Published:** 2016-11-11

**Authors:** D. Avanzato, T. Genova, A. Fiorio Pla, M. Bernardini, S. Bianco, B. Bussolati, D. Mancardi, E. Giraudo, F. Maione, P. Cassoni, I. Castellano, L. Munaron

Scientific Reports 6: Article number: 3260210.1038/srep32602; published online: 09 02 2016; updated: 11 11 2016

The original version of this Article contained typographical errors in the Abstract.

In the HTML version of the published Article

“Lower doses (1–10 mm result ineffective”

now reads:

“Lower doses (1–10 μM) result ineffective”

In the PDF version of the Article

“Lower doses (1–10 μm result ineffective”

now reads:

“Lower doses (1–10 μM) result ineffective”

These errors have now been corrected in both the PDF and HTML versions of the Article.

