# Supplementary material for: Activation of P2X7 and P2Y11 purinergic receptors inhibits migration and normalizes tumor-derived endothelial cells via cAMP signaling
Source: Sci Rep. 2016 Sep 2;6:32602. doi: 10.1038/srep32602 (PMC5009337; doi:10.1038/srep32602)
Supplement: Supplementary Information [file srep32602-s1.pdf]

Activation of P2X7 and P2Y11 purinergic receptors inhibits migration and normalizes tumor-derived endothelial cells *via* cAMP signaling.

D. Avanzato[1], T. Genova[1], A. Fiorio Pla[1,2,] M. Bernardini[1], S. Bianco[1], B. Bussolati[3], D. Mancardi[4], E. Giraudo[5], F. Maione[5], P. Cassoni[6], I. Castellano[6] & L. Munaron[1,2]

[1]Department of Life Sciences & Systems Biology, [2]Nanostructured Interfaces and Surfaces Centre of Excellence (NIS), University of Torino, Italy, [3] Dept. of Molecular Biotechnology and Health Sciences, University of Torino, Italy, [4]Department of Clinical and Biological Sciences, University of Turin, Italy, [5]Candiolo Cancer Research Center, Torino, Italy [6]Department of Medical Sciences, Torino, Italy.

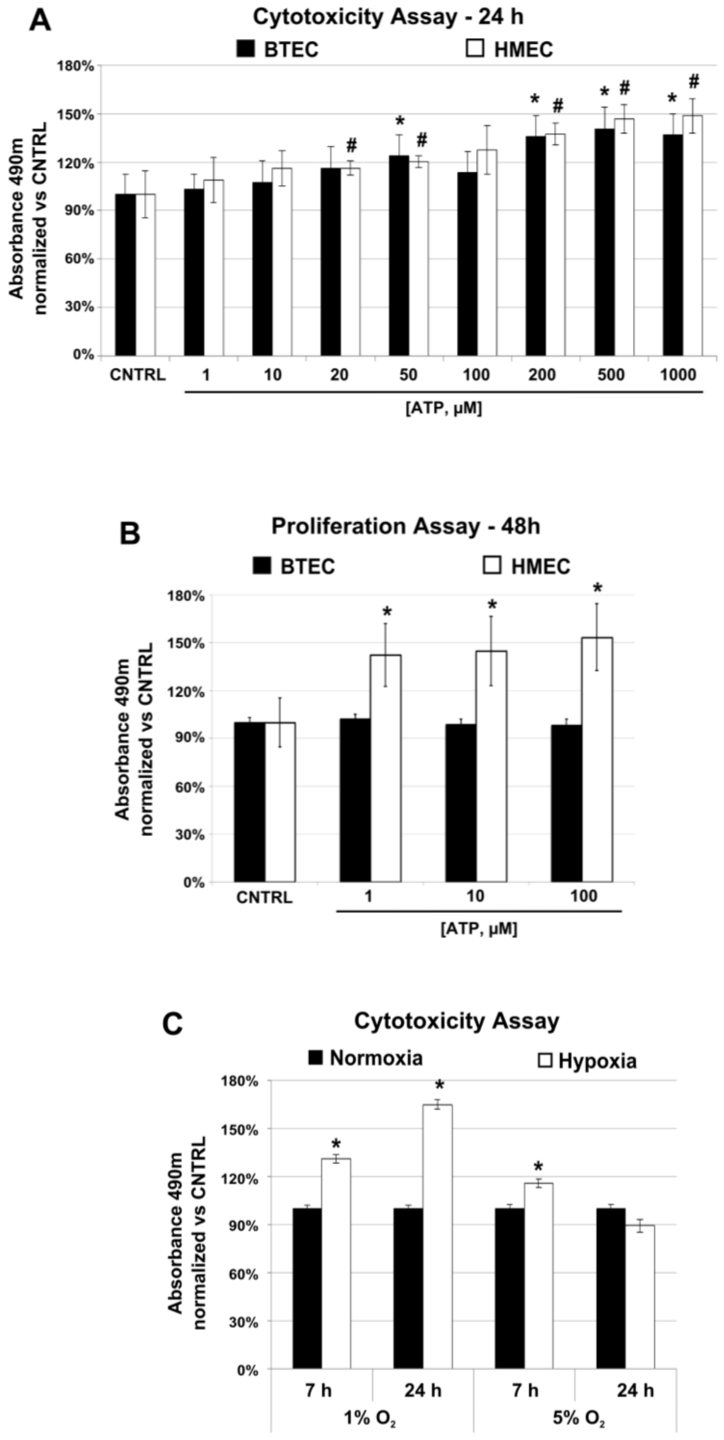

D

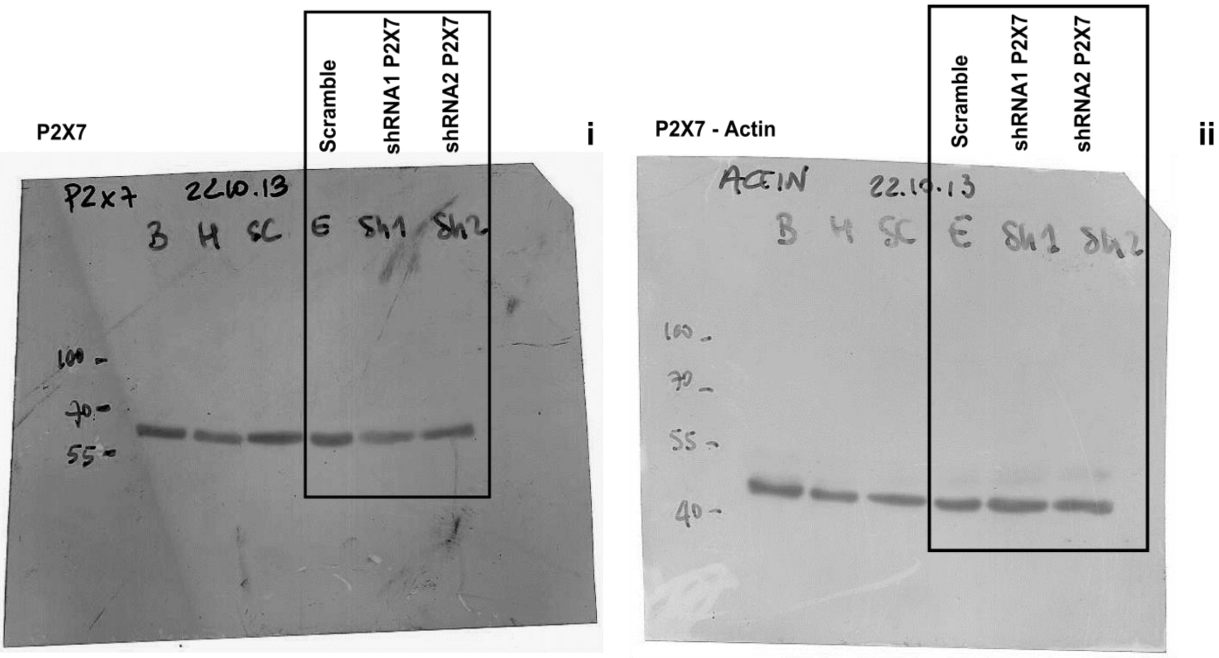

E

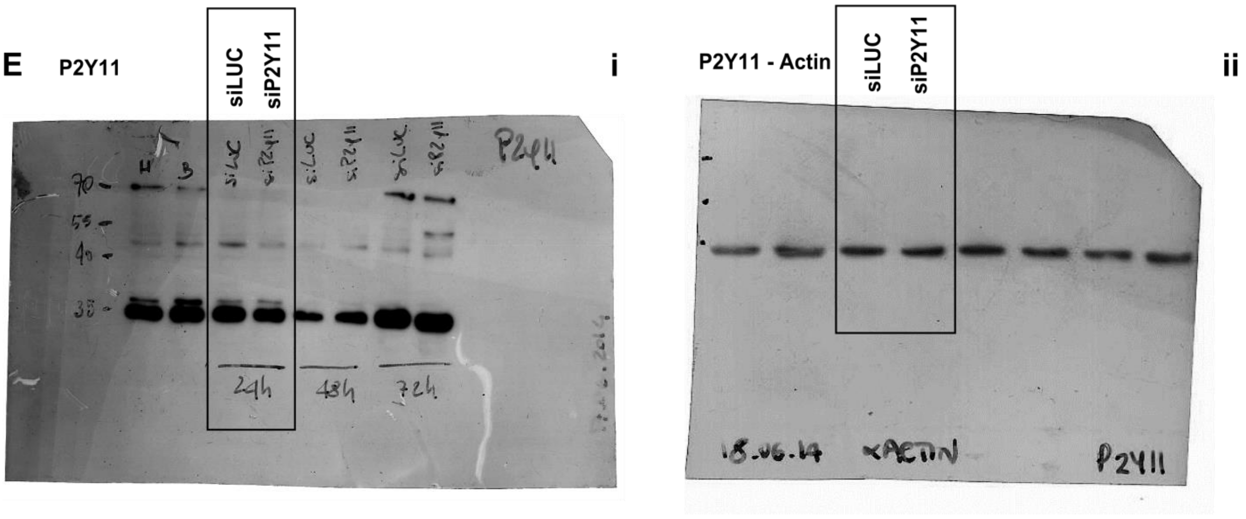

Cytotoxicity at 24 hrs with different ATP concentrations (**A**) and proliferation at 48 hrs (**B**) evaluated by MTS assay; data were normalized to the control (CNTRL) for each cell type: BTEC (black bars) and HMEC (white bars). Data from three independent experiments are expressed as mean ± S.E.M. Wilcoxon test: *p<0.001 vs. BTEC CNTRL; #p<0.001 vs. HMEC CNTRL.
Cytotoxicity at 7 and 24 hrs in BTEC maintained in normoxic and hypoxic (1% and 5% O_2 as reported) conditions evaluated by MTS assay; data were normalized to the control (CNTRL) for each time and condition. Data from three independent experiments are expressed as mean ± S.E.M. Wilcoxon test: *p<0.001 vs. corresponding CNTRL (**C**).
Full-length Western Blot for P2X7 (**D**) and P2Y11 (**E**). Blot of P2X7 (**D,i**) and relative Actin (**D,ii**) antibody decoration are shown. In the same way P2Y11 (**E,i**) and relative Actin (**E,ii**) are reported. Actin staining was performed on the same blot previously decorated with P2X7 or P2Y11 antibody.
In Figure 3 we cropped the selected lines (black box) of these original files.

Appendix.

Compound	Chemical name	Concentrations	Molecular implications
**ATP**	*Adenosine 5'-trisphosphate*	1, 10, 100 µM	P2X and P2Y agonist
**ADP**	*Adenosine 5'-diphosphate*	100 µM	P2Y1/12/13 agonist
**UTP**	*Uridine 5'-triphosphate*	100 µM	P2Y2/4/6 agonist
**ADO**	*Adenosine*	100 µM	P1 receptor agonist
**ATP-γ-S**	*Adenosine-5'-(γ-thio)-triphosphate*	100 µM	Non-hydrolyzable analog of ATP
**CGS 15943**	*9-Chloro-2-(2-furanyl)-[1,2,4]triaz-olo[1,5-c]quinazolin-5-amine*	1 µM	Adenosine receptor (P1R) antagonist
**FK (Forskolin)**	*7β-Acetoxy-8,13-epoxy-1α,6β,9α-trihydroxylabd-14-en-11-one*	10 µM	Adenylyl cyclase (AC) activator
**BzATP**	*2'(3')-O-(4-Benzoylbenzoyl)adenosine 5'-triphosphate*	100 µM	P2X7 and P2Y11 activator
**CPA**	*α-cyclopiazonic acid*	30 µM	Specific inhibitor of Ca^{2+}-ATPase
**8-Br-cAMP**	*8-Bromoadenosine-3',5'-cyclic monophosphate*	500 µM	cAMP analogue
**IBMX**	*1-Methyl-3-isobutylxanthine*	100 µM	Phosphodiesterase inhibitor
**8-CPT (2'-O-Me-cAMP)**	*8-(4-Chlorophenylthio)-2'-O-methyladenosine-3',5'-cyclic monophosphate*	100 µM	Epac-1 activator
**H89**	*N-[2-(p-Bromocinnamylamino)ethyl]-5-isoquinolinesulfonamide dihydrochloride*	10 µM	inhibitor of cAMP-dependent protein kinase A (PKA)
**NF157**	*8,8'-[Carbonylbis[imino-3,1-phenylenecarbonylimino(4-fluoro-3,1-phenylene)carbonylimino]]bis-1,3,5-naphthalenetrisulfonic acid*	50 µM	P2Y11 antagonist
**ddAdo**	*2',5'-Dideoxyadenosine*	10 µM, 1 mM	Adenylyl cyclase inhibitor
**$NaHCO_3$**	*Sodium Bicarbonate*	25 mM	selective activator of AC10
**BBG**	*Brilliant Blue G*	20 µM	P2X7 antagonist
